# Anxiety mediates the effect of social media addiction on negative attentional bias: the moderating role of impulsivity

**DOI:** 10.3389/fpsyt.2025.1592132

**Published:** 2025-06-27

**Authors:** Rui Qiu, Yushan Li, Yue Gong, Zhihua Guo, Sizhe Cheng, Mengze Li, Xia Zhu

**Affiliations:** Department of Military Medical Psychology, Air Force Medical University, Xi’an, China

**Keywords:** social media addiction, negative attentional bias, anxiety, impulsivity, moderated mediation model

## Abstract

**Objective:**

In China, platforms such as WeChat serve as integral hubs for communication, education, and daily life, rendering social media addiction a pressing concern among university students. Their profound digital immersion, combined with academic pressures, creates a unique contextual milieu where the cognitive ramifications of addiction, including negative attentional bias may be exacerbated. This study therefore aims to investigate the mediating role of anxiety and the moderating role of impulsivity in the relationship between social media addiction and negative attentional bias.

**Methods:**

A cross-sectional survey was conducted with 1,006 Chinese university students (81.1% male; the mean age of participants was 21.45 ± 2.013 years). Participants completed measures of social media addiction, anxiety, negative attentional bias, and impulsivity. Data were analyzed using SPSS and PROCESS macros for mediation and moderation effects with bootstrapping.

**Results:**

Social media addiction directly predicted negative attentional bias (*β* = 0.270, *p* < 0.001) and indirectly through anxiety (indirect effect = 0.111, 95% CI [0.073, 0.153]). Impulsivity moderated both the direct effect (*β* = -0.020, *p* < 0.001) and the anxiety-mediated pathway (*β* = -0.026, *p* < 0.001). Specifically, anxiety strongly predicted negative attentional bias at low impulsivity (*β* = 0.893, *p* < 0.001) but not at high impulsivity (*β* = 0.023, *p* = 0.730).

**Conclusion:**

his study reveals a moderated mediation model where anxiety mediates the effect of social media addiction on negative attentional bias, and impulsivity buffers this relationship. These findings highlight the importance of addressing anxiety and impulsivity in interventions for social media addiction-related cognitive biases.

## 1 Introduction

With the rapid development of information technology and the widespread use of social media, profound changes are occurring in people’s lifestyles (1). Social media has not only altered the way people access information but has also reshaped their cognitive patterns, emotional processing mechanisms, and social interaction behaviors (2). Although social media plays a significant role in information dissemination and social connectivity, its addictive characteristics are increasingly becoming a concern (3). Studies have shown that social media addiction is closely associated with various psychological issues, including anxiety, depression, and other mental health problems (4, 5).

Social media addiction is defined as an individual’s excessive reliance on social media, characterized by difficulties in controlling usage frequency and strong reactions to withdrawal in terms of cognition, behavior, and physiology (6). This excessive use leads to a series of negative consequences, causing varying degrees of damage to individuals in multiple critical areas of life, including social activities, interpersonal relationship building and maintenance, learning and work efficiency, physical and mental health, and overall well-being (7).

In terms of social activities, addicted individuals may reduce the frequency and quality of their participation in real-life social events due to excessive indulgence in virtual socializing (8). On the interpersonal level, over-reliance on social media interaction can lead to alienation and a decline in the quality of real-life relationships (9). Regarding learning and work, spending a significant amount of time on social media reduces individuals’ focus and energy on studying and working, thereby affecting academic performance and job efficiency (10). In the health domain, poor posture resulting from long-term social media use can cause musculoskeletal problems such as neck and back pain. Additionally, excessive use can disrupt normal sleep patterns, affecting physical and mental health (11). Ignoring important activities and relationships in real life can also negatively impact individuals’ life satisfaction and happiness (7).

The causes of social media addiction are complex and multifaceted. From an individual psychological perspective, individuals with a stronger sense of loneliness often seek social support and emotional resonance on social media, attempting to fill the void left by real-life social interactions (11). Those with low self-esteem hope to enhance their sense of self-worth through positive feedback such as likes and comments on social media. This excessive reliance on external validation can easily lead to addictive behaviors (10). In terms of social environmental factors, the influence of peers is significant. When frequent social media use is prevalent within an individual’s social circle, the individual may increase their own usage frequency due to conformity, gradually leading to addiction (12). Furthermore, social media platforms’ algorithm-based content recommendation systems and instant interactive feedback mechanisms, such as likes, comments, and push notifications, constantly stimulate users’ brain reward systems. This increases dopamine secretion, reinforces user behavior, and makes users more likely to become addicted (13).

Research indicates that social media addiction can trigger a series of adverse consequences. In the field of mental health, it is closely linked to various psychological issues such as depression and anxiety. Studies have found that social media addicts score significantly higher on depression scales compared to non-addicts (14). Other research suggests a significant positive correlation between social media addiction and anxiety symptoms (15). On the cognitive level, social media addiction potentially impacts individuals’ attention, memory, thinking ability, and other cognitive functions (16). Becker et al. pointed out that social media addiction may distract individuals and interfere with effective information processing and storage (17).

Negative attentional bias refers to an individual’s tendency to prioritize attention and processing of negative information over neutral or positive information when faced with multiple stimuli (18). A high level of negative attentional bias can make individuals more susceptible to negative emotional states, increasing the risk of psychological disorders (19). Studies have shown that negative attentional bias plays a crucial role in the development and maintenance of psychological illnesses such as anxiety and depression (20). Therefore, exploring the relationship between social media addiction and negative attentional bias holds significant theoretical and practical importance.

### 1.1 The impact of social media addiction on negative attentional bias

Social media addiction may reshape an individual’s cognitive patterns and attentional allocation mechanisms (21). With long-term immersion in the social media environment, individuals frequently encounter a vast amount of complex information. Negative information, due to its stronger emotional arousal, may lead individuals to gradually develop an attentional preference for such information, resulting in a negative attentional bias (15, 18).

However, current research on the specific ways social media addiction affects negative attentional bias, the extent of its impact, and the underlying psychological and neural mechanisms remains relatively scarce and requires further exploration.

### 1.2 The mediating effect of anxiety between social media addiction and negative attentional bias

Anxiety may play a mediating role between social media addiction and negative attentional bias. On the one hand, studies have shown that social media addiction is closely related to the generation of anxiety. Factors such as social comparison, information overload, and privacy concerns during social media use can significantly increase an individual’s anxiety level (22). On social media platforms, people often unconsciously engage in social comparison, and when they find themselves lacking in certain aspects compared to others, they are prone to negative emotions such as self-doubt and anxiety. Information overload can also cause individuals to feel stressed and anxious when processing large amounts of information (23). On the other hand, anxiety significantly affects an individual’s cognitive processing, especially in terms of attentional allocation (24). Individuals in a state of anxiety tend to prioritize their cognitive resources towards negative information, and this attentional bias further reinforces anxiety, creating a vicious cycle (24).

This study hypothesizes that anxiety plays a mediating role between social media addiction and negative attentional bias. In other words, social media addiction leads to an increase in anxiety levels, which in turn enhances negative attentional bias.

### 1.3 The moderating effect of impulsivity between anxiety and negative attentional bias

Impulsivity refers to an individual’s tendency to respond quickly and without much deliberation when faced with stimuli, often accompanied by weaker self-control abilities (25). Individuals with higher impulsivity may find it more difficult to inhibit their attention to negative information when facing anxiety, due to their relatively weaker self-regulation and cognitive control abilities. This may exacerbate negative attentional bias (25). Studies have shown that impulsivity affects individuals’ regulation of emotions and cognition, and those with high impulsivity are more prone to cognitive biases and behavioral loss of control when faced with emotional stimuli (26). Conversely, individuals with lower impulsivity often have better emotion regulation and cognitive control strategies. When facing anxiety, they can more effectively adjust their attention and reduce excessive focus on negative information, making the impact of anxiety on negative attentional bias relatively weaker (26). Research shows that individuals with low impulsivity perform better in cognitive reappraisal tasks and can better adjust their attention to negative information.

This study speculates that impulsivity plays a moderating role between anxiety and negative attentional bias, meaning that the level of impulsivity can affect the strength of anxiety’s influence on negative attentional bias.

### 1.4 Research hypotheses

Based on the theoretical framework and literature review, this study proposes the following specific hypotheses to be tested.

Social media addiction will indirectly predict negative attentional bias through anxiety. Specifically, higher levels of social media addiction will increase anxiety, which in turn will strengthen negative attentional bias.

*Hypothesis 1: Anxiety mediates the relationship between social media addiction and negative attentional bias.*

Impulsivity will moderate the direct effect of social media addiction on negative attentional bias. The positive association between social media addiction and negative attentional bias will be stronger among individuals with higher impulsivity, due to their reduced inhibitory control and heightened susceptibility to impulsive information processing.

*Hypothesis 2: Impulsivity moderates the direct effect of social media addiction on negative attentional bias.*

Impulsivity will also moderate the anxiety-mediated pathway. The positive effect of anxiety on negative attentional bias will be more pronounced among individuals with lower impulsivity, as their better cognitive control allows anxiety to more effectively drive attentional prioritization of negative information. Conversely, this effect will be weaker among those with higher impulsivity, who may exhibit less sustained attention to negative stimuli despite elevated anxiety.

*Hypothesis 3: Impulsivity moderates the mediating effect of anxiety in the relationship between social media addiction and negative attentional bias*.

These hypotheses collectively form a moderated mediation model, aiming to clarify how anxiety acts as a psychological bridge between social media addiction and cognitive bias, and how individual differences in impulsivity shape this pathway. Testing these hypotheses will deepen our understanding of the complex interplay between addictive behaviors, emotional states, dispositional traits, and cognitive processing in the digital age.

In summary, this study intends to construct a moderated mediation model to deeply explore the impact mechanism of social media addiction on negative attentional bias, examining the mediating effect of anxiety and the moderating effect of impulsivity. The aim is to untangle the pathway of social media addiction’s influence on negative attentional bias and clarify the mediating role of anxiety and the moderating role of impulsivity. Theoretically, this contributes to enriching and expanding the theoretical framework in the field of social media addiction, further improving the research framework for the impact of addictive behaviors on cognitive processing mechanisms, and providing a more solid theoretical foundation for subsequent related studies. Practically, the findings of this study can provide a strong basis for developing scientifically effective intervention measures. By deeply understanding these impact mechanisms, personalized psychological intervention programs can be designed for social media addicts with different traits. Simultaneously, prevention strategies can be developed based on the research results to enhance self-management awareness and ability in social media use, prevent addictive behaviors and their associated negative impacts, and promote individual mental health and comprehensive development.

## 2 Materials and methods

### 2.1 Participants

A convenient sampling method was employed to conduct an online survey among university students using a questionnaire platform. A total of 1006 valid questionnaires were collected. Among them, 816 were male (81.1%), and 190 were female (18.9%). The participants’ ages ranged from 17 to 26 years, with an average age of 21.45 ± 2.013 years. Seven participants (0.7%) had junior high school education or below, 430 (42.7%) had high school or vocational education, 563 (56.0%) had a university degree, and 6 (0.6%) had a postgraduate degree or above. The average daily mobile phone usage was 3.93 ± 3.286 hours.

### 2.2 Measurement tools

#### 2.2.1 Bergen Social Media Addiction Scale

In this study, the Bergen Social Media Addiction Scale (BSMAS) was used to evaluate individuals’ levels of addiction to social media (27, 28). The scale consists of six items rated on a 5-point Likert scale (1 = very rarely, 5 = very often). Each item describes a dimension of addictive behavior: salience, mood modification, tolerance, withdrawal symptoms, conflict, and relapse.

Some topics of the scale are as follows: “I spend more time on social media than I intended.” “I feel anxious or restless when I cannot use social media.” The total score of the BSMAS ranges from 6 to 30, with higher scores indicating a higher degree of social media addiction. In this study, the Cronbach’s alpha for the scale was 0.914.

#### 2.2.2 Generalized Anxiety Disorder Scale

The Generalized Anxiety Disorder Scale (GAD-7) was used to screen and evaluate symptoms of generalized anxiety in this study (29). The scale consists of 7 items rated on a scale from 0 (not at all) to 3 (nearly every day). The total score ranges from 0 to 21, with scores of 0–4 indicating no anxiety, 5–9 indicating mild anxiety, 10–14 indicating moderate anxiety, and 15–21 indicating severe anxiety. Higher scores indicate higher levels of anxiety. In this study, the Cronbach’s alpha for the scale was 0.944. Some topics of the scale are as follows: “Have you been feeling nervous, anxious, or on edge?” “Have you been unable to stop or control worrying?”

#### 2.2.3 Attention to Positive and Negative Information Scale - Negative Information Attention Subscale

The Negative Information Attention Subscale (ANI) from the Attention to Positive and Negative Information Scale (APNI) was used to assess individuals’ attention to negative information in their lives (30, 31). The ANI consists of 10 items rated on a 5-point scale from 1 (completely disagree) to 5 (completely agree). Higher scores indicate a more pronounced attention bias towards negative information. Some topics of the scale are as follows: “I tend to focus more on negative events in my life than positive ones.” “Negative comments from others affect me more than positive ones.” In this study, the Cronbach’s alpha for the subscale was 0.856.

#### 2.2.4 Barratt Impulsiveness Scale

The Chinese revised version of the Barratt Impulsiveness Scale (BIS) was used as an assessment tool for impulsivity in this study (32). The scale includes three subscales: non-planning, motor impulsiveness, and cognitive impulsiveness. Each subscale contains 10 items, with scores ranging from 1 to 5 (never, rarely, sometimes, often, and always). Higher scores indicate higher impulsivity. Some topics of the scale are as follows: “I often do things without planning.” “I act on impulse without thinking.” “I have trouble concentrating on complex tasks.” In this study, the Cronbach’s alpha for the scale was 0.935.

### 2.3 Data processing and analysis

Statistical analyses were conducted using SPSS 29.0 and PROCESS 4.0, with a two-tailed p-value threshold of < 0.05 for statistical significance. Continuous variables (social media addiction, anxiety, negative attentional bias, impulsivity) were summarized using means (M), standard deviations (SD), and skewness/kurtosis values to assess normality. Categorical variables (gender, education level) were described using frequencies (n) and percentages (%). All continuous variables were standardized to reduce multicollinearity. A nonparametric bootstrap procedure with 5,000 resamples was used to estimate 95% bias-corrected confidence intervals (CIs) for indirect effects and conditional effects at moderator levels. Overall fit was evaluated using R² (proportion of variance explained) and F-tests. For the full moderated mediation model. The indirect effect’s significance was determined by bootstrapped CIs; if the CI does not include zero, the effect is considered significant.

### 2.4 Ethical considerations

Prior to data collection, all participants provided written informed consent via an online form embedded in the survey platform. The consent document detailed key ethical considerations: (1) Study Objectives, including a clear description of the research purpose (investigating social media addiction, anxiety, impulsivity, and negative attentional bias), measured variables, and expected participation duration; (2) Voluntary Participation, emphasizing that involvement was optional with the right to withdraw at any time without consequences or loss of entitlements; (3) Confidentiality Measures, ensuring data anonymization through unique participant ID numbers (instead of personal identifiers).

Given the study’s non-invasive nature and the IRB’s determination of exemption from formal ethical review due to minimal risk, ongoing ethical monitoring was waived. No adverse events were reported during or after data collection.

## 3 Results

### 3.1 Common method bias test

In this study, Harman’s one-factor analysis was adopted to test for the presence of common method bias (33). The results indicated that there were seven factors with eigenvalues greater than 1, and the variance explained by the first factor was 17.37%, which is below the critical value of 40%. This suggests a low possibility of common method bias in the current study.

### 3.2 Correlation analysis of social media addiction, anxiety, and attentional bias

A correlation analysis was conducted among scores of social media addiction, anxiety, attentional bias, and their respective dimensions. SPSS statistical testing revealed significant positive correlations between social media addiction and anxiety, negative attentional bias, as well as impulsivity (p<0.01). Specifically, higher scores of social media addiction were associated with higher scores of anxiety, negative attentional bias, and impulsivity. Moreover, significant positive correlations were observed among all four variables: social media addiction, anxiety, negative attentional bias, and impulsivity (*p*<0.01). Detailed descriptive statistics and the correlation matrix are presented in **Table 1**.

**Table 1:** Mean, standard deviation and correlation coefficient of each variable (*n*=1006).

Variable	Mean	Standard deviation	1	2	3	4	5
1. social media addiction	12.80	5.05	1				
2. anxiety	10.38	4.02	0.47^**^	1			
3. positive attention bias	44.48	7.95	-0.12^**^	-0.23^**^	1		
4. negative attentional bias	30.98	6.76	0.35^**^	0.30^**^	0.34^**^	1	
5. impulsivity	71.65	16.62	0.41^**^	0.50^**^	-0.48^**^	0.26^**^	1

^**^means *p<*0.01.

### 3.3 Examination of the mediating effect of anxiety between social media addiction and negative attentional bias

The prerequisite for mediation effect testing is that the independent variable, dependent variable, and mediating variable should all have significant pairwise correlations. Therefore, this study meets the prerequisite for testing the mediation effect, as significant pairwise correlations exist between the three variables: social media addiction, anxiety, and negative attentional bias.

**Figure 1:**
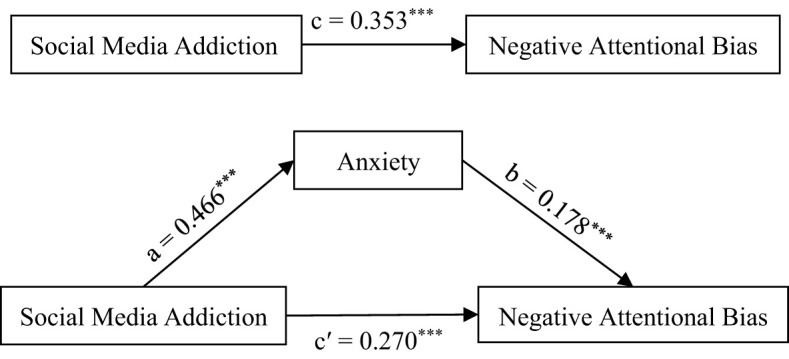
Mediating model of social media addiction, anxiety and negative attention bias. *** means *P*<0.001.

In this study, we employed a combination of stepwise regression equations and the Process method to analyze the mediation effect. The main steps are as follows: Firstly, we used social media addiction as the predictor variable and negative attentional bias as the dependent variable to test the predictive effect of social media addiction on negative attentional bias. Secondly, we considered social media addiction as the predictor variable and anxiety as the dependent variable to examine the predictive effect of social media addiction on anxiety. Finally, we took both social media addiction and anxiety as predictor variables, and negative attentional bias as the dependent variable, to investigate the combined predictive effect of social media addiction and anxiety on negative attentional bias. Specific results are presented in **Table 2** and **Figure 1**.

**Table 2:** The mediating effect of anxiety on social media addiction and negative attention bias.

	Predictor	Dependent variable	*R* ^2^	Adjust *R* ^2^	*F*	*β*	*t*
1. Path c	social media addiction	negative attentional bias	0.124	0.124	142.617^***^	0.353	11.942^***^
2. Path a	social media addiction	anxiety	0.217	0.216	278.657^***^	0.466	16.693^***^
3.Path b, c′	anxiety	negative attentional bias	0.149	0.148	87.974^***^	0.178	5.414^***^
	social media addiction					0.270	8.190^***^

^***^means *p<*0.001.

According to **Table 2** and **Figure 1**, social media addiction has a significant positive predictive effect on negative attentional bias (c = 0.353, *p* < 0.001) and a significant positive predictive effect on anxiety (a = 0.466, *p* < 0.001). When both social media addiction and anxiety are included in the regression equation, anxiety has a significant positive predictive effect on negative attentional bias (b = 0.178, *p* < 0.001), and social media addiction still maintains a significant positive predictive effect on negative attentional bias (c’ = 0.270, *p* < 0.001). Based on the above data analysis results, it is indicated that anxiety plays a mediating role between social media addiction and negative attentional bias, with the mediating effect accounting for 23.50% of the total effect (a*b/c).

To further verify whether the mediating effect of anxiety is statistically significant, this study used the simple mediation model of the Process plugin in SPSS. The Bootstrap method was adopted to test the mediating effect of anxiety between social media addiction and negative attentional bias. With social media addiction as the independent variable, negative attentional bias as the dependent variable, and anxiety as the mediating variable, Model 4 in the Process plugin was used with a sample size of 5000 to evaluate the 95% confidence interval. If the confidence interval does not include 0, it indicates a significant mediating effect. The results are shown in **Table 3**.

**Table 3:** The mediating effect of anxiety on social media addiction and negative attention bias.

	Effect size	Boot SE	Boot LLCI	Boot ULCI	Effect ratio
Indirect effect	0.1112	0.0205	0.0726	0.1528	23.56%
Direct effect	0.3608	0.0441	0.2744	0.4473	76.44%
Total effect	0.472	0.0395	0.3945	0.5496	

According to **Table 3**, the indirect effect of anxiety predicting negative attentional bias through social media addiction is 0.111, and the 95% confidence interval is [0.073, 0.153]. This interval does not include 0, verifying that the mediating effect of anxiety is statistically significant. Therefore, anxiety plays a significant mediating role between social media addiction and negative attentional bias.

### 3.4 Examination of the moderating effect of impulsivity on the relationship between anxiety and negative attentional bias

Using PROCESS Model 14, the moderating effect of impulsivity was tested. Prior to analysis, all variables were standardized. The results indicated a significant predictive effect of the interaction between impulsivity and anxiety on negative attentional bias (*β* = -0.026, SE = 0.004, *p* < 0.001), suggesting that impulsivity can moderate the relationship between anxiety and negative attentional bias. The findings are summarized in **Table 4**.

**Table 4:** Test of the regulating effect of impulsivity.

Regression equation		Overall fit index			Significance of regression coefficient	
Outcome	Predictor	*R*	*R* ^2^	*F*	*β*	*t*
negative attentional bias	social media addiction	0.435	0.189	58.488^***^	0.324	7.302^***^
	anxiety				0.458	6.836^***^
	impulsivity				0	-0.020
	anxiety x impulsivity				-0.026	-6.595^***^

All continuous variables in the model are standardized and brought into the regression equation.

^***^means *p<*0.001.

**Figure 2:**
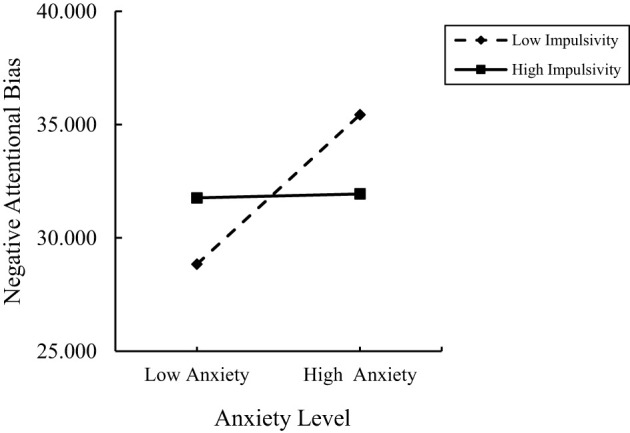
The influence of anxiety on negative attention bias at different levels of impulsivity.

To further elucidate the nature of the interaction between anxiety and impulsivity, anxiety and impulsivity scores were grouped into high and low categories based on one standard deviation above and below the mean, respectively. A simple slope test was conducted, and the results are illustrated in **Figure 2**. When impulsivity levels were high (M+1SD), the predictive effect of anxiety on negative attentional bias was weaker (*β* = 0.023, *t* = 0.345, *p* = 0.730). Conversely, when impulsivity levels were low (M-1SD), the predictive effect of anxiety on negative attentional bias was significantly stronger (*β* = 0.893, *t* = 7.798, *p* < 0.001).

## 4 Discussion

This study examines the complex interplay between social media addiction, anxiety, impulsivity, and negative attentional bias among Chinese university students, revealing a moderated mediation model where anxiety mediates the effect of social media addiction on negative attentional bias, and impulsivity buffers this relationship. These findings contribute to both theoretical understanding and practical interventions in the context of digital addiction and cognitive bias.

### 4.1 Theoretical contributions: elaborating the psychological mechanisms

On the theoretical level, our finding that anxiety significantly mediates between social media addiction and negative attentional bias echoes previous research highlighting anxiety’s mediating role in various psychological associations (22). This further enriches the theoretical framework linking addictive behaviors and cognitive processing. Social media addicts are constantly exposed to a complex information environment, where frequent stimulation by negative information can easily trigger anxiety. In an anxious state, individuals tend to prioritize cognitive resources towards negative information, leading to a negative attentional bias (34). For instance, the common “perfect life” presentations on social media can induce self-doubt and anxiety in addicts through unconscious social comparison, resulting in a heightened focus on negative information and a vicious cycle of negative emotions (34).

### 4.2 Core findings: anxiety as a mediator and impulsivity as a moderator

This not only elucidates the underlying psychological process of how social media addiction affects negative attentional bias but also provides empirical evidence for related theoretical studies. However, it’s noteworthy that anxiety’s mediating effect only accounts for 23.5% of the total effect, indicating the presence of other unidentified pathways influencing negative attentional bias in social media addiction. Future research could explore the neural mechanisms using functional magnetic resonance imaging (fMRI) to observe differences in brain activity when addicts process information with varying emotional valence. This could help uncover the neural pathways through which addiction affects negative attentional bias, refining the theoretical framework and offering a more comprehensive explanation of this complex psychological phenomenon. Regarding moderation effects, impulsivity’s moderating role between anxiety and negative attentional bias provides new insights into understanding individual differences in the development of psychological issues. Highly impulsive individuals find it difficult to suppress their attention to negative information during anxiety, weakening the impact of anxiety on negative attentional bias. Conversely, less impulsive individuals exhibit better attentional control, significantly enhancing the effect of anxiety on negative attentional bias (35). For instance, in similar social media usage scenarios and anxiety-inducing situations, highly impulsive individuals may be briefly attracted to negative information but quickly shift their focus, while less impulsive individuals tend to ruminate on negative information, reinforcing negative attentional bias and leading to more severe negative emotions (36). This discovery underscores the importance of considering individual differences in impulsivity when designing psychological intervention programs. For highly impulsive social media addicts, apart from training in emotion regulation and cognitive control skills, they can be guided to utilize their ability to quickly shift attention to reduce the impact of negative information. For less impulsive individuals, the focus should be on helping them break the pattern of excessive focus on negative information, providing theoretical guidance for personalized interventions.

### 4.3 Limitations and future directions

While this study advances our understanding of the moderated mediation model linking social media addiction to negative attentional bias, several limitations warrant attention. In terms of the sample, despite a sample size of 1006, it’s important to note that social media usage habits, perceptions of addiction, and ways of coping with anxiety can vary across different cultural backgrounds. In collectivist cultures, individuals tend to prioritize group harmony, and social media usage may be more geared towards maintaining group relationships. As a result, manifestations and consequences of addiction might differ from those in individualistic cultures, and the relationship between anxiety and negative attentional bias could also be influenced by cultural values (37).

The cross-sectional design precludes causal inference, as we cannot determine the temporal ordering of variables. The sample comprised primarily male students from a Chinese military academy, limiting generalizability to diverse cultural, gender, and institutional contexts. Reliance on self-report measures may introduce response bias, despite robust reliability evidence. To address these limitations, future research could adopt longitudinal designs to clarify causal pathways. For example, could use cross-lagged panel models to test whether increases in social media addiction predict subsequent anxiety (Path a) and whether anxiety, in turn, predicts heightened negative attentional bias (Path b), with impulsivity tested as a time-stable moderator. Such designs would resolve the cross-sectional limitation by establishing temporal precedence.

The gender disparity in our sample (81.1% male), while reflective of the military academy’s demographic composition, restricts the generalizability of our findings to populations with balanced sex distributions. Gendered media ecology theories suggest that sex-related differences in social media motivations and regulatory processes may moderate the anxiety-mediated pathway, an aspect underdetermined in this study. Future research should prioritize representative sampling and multigroup modeling to elucidate how gender influences the cognitive consequences of social media addiction, thereby enhancing the framework’s generalizability. Hence, conducting cross-cultural studies to compare the similarities and differences in these relationships among different cultural groups can enhance the universality and applicability of research findings. Regarding measurement methods, all variables in this study were based on self-reports, which can be influenced by individual subjective perceptions and memory biases, potentially leading to measurement errors. Future research could incorporate objective methods such as behavioral observation and physiological measurements, including recording actual behavioral data during social media usage and measuring physiological indicators like skin conductance response and heart rate variability to reflect anxiety levels. This would improve the accuracy of the study and reveal the relationships between variables more objectively.

While our model posits anxiety as a key mediator, several alternative frameworks warrant consideration. First, unmeasured individual differences such as self-esteem and social support may confound the observed associations; for example, low self-esteem could drive both excessive social media use and heightened sensitivity to negative social cues (38, 39). Second, constructs like fear of missing out (FoMO) or cognitive rumination may offer competing mediational pathways, as FoMO has been linked to attentional hypervigilance toward social threats independent of anxiety (40). Methodologically, the cross-sectional design precludes causal inference, and unmeasured third variables (e.g., personality traits, self-regulatory capacity) may account for the observed relationships. These limitations highlight the need for future research to incorporate longitudinal designs, multi-source data, and expanded mediational models to disentangle these complex mechanisms (41).

On the intervention front, findings suggest targeting anxiety and impulsivity in tailored programs. A randomized controlled trial could evaluate a cognitive-behavioral intervention integrating anxiety regulation techniques and impulsivity control strategies, delivered via mobile apps to enhance accessibility (42, 43). Participants would be stratified by impulsivity levels to test whether efficacy varies across subgroups, aligning with our moderation findings. Additionally, cross-cultural replication studies in non-military samples with balanced gender distributions would strengthen generalizability, using multigroup structural equation modeling to compare pathway invariance across contexts (44).

In summary, this study provides a new perspective for understanding the relationships between social media addiction, anxiety, impulsivity, and negative attentional bias through mediation and moderation effect analysis. Future research should continuously explore other potential influencing factors and mechanisms (45), adopting more rigorous research designs and methods, such as multivariable crossover studies and longitudinal tracking studies, to provide a more scientific and effective basis for preventing and intervening in psychological issues related to social media addiction.

## 5 Conclusion

1. Social media addiction has a significant direct predictive effect on negative attentional bias and can indirectly predict negative attentional bias through the mediating effect of anxiety.
2. Both the direct effect of social media addiction on negative attentional bias and the mediating effect of anxiety are moderated by impulsivity. Compared to individuals with low impulsivity, the direct effect of social media addiction on negative attentional bias is stronger in individuals with high impulsivity.
3. Impulsivity plays a moderating role in the mediating effect of anxiety on negative attentional bias. Compared to individuals with low impulsivity, the mediating effect of anxiety on negative attentional bias is weaker in individuals with high impulsivity.

## Data Availability

The raw data supporting the conclusions of this article will be made available by the authors, without undue reservation.
